# Sport-for-employability as an innovative practice in addressing youth underemployment in sub-Saharan Africa

**DOI:** 10.3389/fspor.2022.1001435

**Published:** 2023-02-06

**Authors:** Cora Burnett

**Affiliations:** Department of Sport and Movement Studies, UJ Olympic Studies Centre, University of Johannesburg, Johannesburg, South Africa

**Keywords:** youth, employability, sport-for-development, nongovernment organizations, sub-Saharan Africa

## Abstract

Youth unemployment reflects the lack of economic growth and prosperity for many nations in sub-Saharan Africa where the average age of the population is estimated to be 24 years in 2050. The study generates insights informed by preliminary findings of an ambitious 6-month government-funded “youth employment program” in South Africa. The paper reports on qualitative data emanating from two field visits per organization providing the baseline of this national project as the researcher had been tasked to develop the monitoring, evaluation, and learning (MEL) system. About 4,000 of this population are from Special Olympics and comprise of youth with complex needs, while the baseline focus on 45 youth as part of the unified leadership initiative (partnering with a mainstream individual). The MEL system allows for a mixed-method approach, but the paper draws on narrative data obtained during field visits where 10 managers were interviewed and 47 youth interns (42.5% men and 57.5% women) took part in focus group discussions. The interview and focus group questions explored their work-related histories and profiles, current involvement with a structured program, experiences, perceptions, expectations, and pragmatic recommendations. Emerging themes and analysis report five main themes that refer to (i) personal employment histories, (ii) local embeddedness, (iii) a typology of work, (iv) program benefits, and (v) enablers and goal setting. Contextual realities shaped the agency of all research participants, but promising results show positive outcomes of soft skills associated with the level of employability and job-seeking strategies.

## 1. Introduction

In Africa, more than 60% of most countries’ populations are under the age of 25 years featuring an increasing “youth bulge” that represents a demographic liability and stressor that contribute to the intensification of vulnerable employment, high levels of youth unemployment, and potential anarchy (1). According to the Quarterly Labour Force Survey (QLFS), South African youth between the age of 15 and 34 years compute 10 million of whom 7.7 million (75.1%) are not part of the labor force, featuring an unemployment rate of 63.9% compared to a 42.1% unemployment rate for 25- to 34-year-old youth (2).

This situation poses unique challenges for economic growth and poverty reduction with specific reference to the Sustainable Development Goal 8 (Target 8.6) that envisaged the reduction of youth not in employment, education, or training (NEET) (3, 4). Neoliberal reforms and economic growth built on the aid-driven initiatives undermine domestic productivity and employability as in the case of Uganda (5).

The complexity of this phenomenon represents a myriad of interlinking factors weaving an ecology across dimensions of social context, the employment environment, neocolonial demand-driven market forces, and multiple layers of disadvantage of young jobseekers (6). Most vulnerable in a stratified dispensation, where political and cultural capital frame realities, are refugees (7), migrants (8), youth from impoverished households or rural areas, women, people with disabilities or those deemed “socially vulnerable” (9, 10), and those stigmatized or marginalized because of their “complex needs” (11). The situation is exacerbated in some industries, such as sport and leisure, and creative industries or tourism associated with unstable employment (12).

In addressing youth unemployment, critical theorists advocate for “resilience thinking” and deconstructing ecological barriers in co-creating a socioeconomic dispensation where youth may find employment, generate income, or find pragmatic and innovative ways of coping with adversity (13). Community-based interventions and nonformal learning may fill a gap for enhancing employability, incubate social mobility, and accrue valuable social capital (14, 15). Interagency approaches and social capital are integral in youth work practice that may act as “a glue between young people and their communities” and create opportunities for employment and the building of collective assets at the local level (16). The generation of bonding (interpersonal, dense, and functional ties based on trust and reciprocity), bridging (connectivity across social divides), and linking social capital (forging connections with people in power), as conceptualized by Putnam (17), foster networking and generating employability opportunities for youth to escape the “revolving door of unemployment” (16). Bonding social capital thus entails horizontal articulation at the individual and interpersonal level to enhance homogeneity (18–20). As bridging social capital, it ensures access to resources within a network arrangement at the collective or community level (17, 21). Linking social capital provides vertical articulation that transcends social stratification and the mobilization of resources external to that of the local community (22). The World Bank values social capital contributing to community integration and solidarity as an integral resource for socioeconomic prosperity (23).

The nongovernmental organization (NGO) sector has a key role to play in nurturing the potential of disadvantaged youth and place them on a journey toward employment by implementing skill-based approaches, sharpening job-seeking skills, and providing therapeutic interventions and supportive work-related environments (11). This research addresses this gap in the South African context of poverty. This paper reports on an employability program that the Sport for Social Change Network Africa (SSCNA) *via* a government grant implemented as a 6-month placement project. The project provided unemployed youth (aged between 18 and 35 years) with paid internships in five NGOs that use sport to provide services to local communities. This research note uses data from the project's innovative monitoring, evaluation, and learning (MEL) to provide preliminary analysis of context and outcomes.

## 2. Methodology

### 2.1. Research design and approach

The research design emanates from a grounded theoretical framework influenced by the work of Corbin and Strauss (24) to feature a pragmatic philosophical approach. It furthers draw on Charmaz's (25) constructivism grounded theory (CGT) that features a constructivist paradigm for data capturing and interpretation. This preliminary study constitutes a preimpact assessment to be followed by several follow-up assessments at the end of the project and a year later. The explorative and evaluation research followed a descriptive and strategic design to provide contextual understandings of individuals in their social settings (26). Piloting the research protocols and standardizing procedures during MEL training ensured input from research participants at the design phase in alignment with a Participatory Action Research approach (10). Different methods, collecting data from different research participant cohorts (managers and interns), and multisite selection implemented triangulation and different perspectives for data interpretation (27).

### 2.2. Methods and data collection

The interview and focus group questions first established the context, profile of the organization (managers), and job-related information (interns) associated with assigned roles and responsibilities of interns within existing structures and practices. All Chief Executive Officers (CEOs) and one senior manager of the five organizations were interviewed with 10 focus groups with 47 interns—two focus groups per organization. Explorative and evaluative questions followed to capture the current program delivery, management, and uptake within the local community. Perceived benefits for the individual, organization, community, and relevant stakeholders allude to envisaged (and unintended) program outcomes. An assessment of quality coupled with identified good practices and challenges set the scene for recommendations to different stakeholders.

The lead researcher conducted all interviews and focus groups on-site that took about 40–90 min depending on the level of knowledge sharing and size of the focus groups. All research participants signed informed consent and gave written permission for the recording of interviews or discussions. To ensure quality communication, a native speaker translated where necessary, while with the Special Olympics South Africa (SOSA) participant, the facilitator translated to ensure the questions were pitched correctly.

### 2.3. Sample

The researcher interviewed a purposive sample of managers (*n* = 10) of the implementing organizations and conducted a focus group session with interns (excluding at one site that served as a training center for SOSA where interviews were conducted with two project managers and one intern in the carpentry section) (28). In the focus group discussions at their community facilities or at the head offices of SOSA and Children of the Dawn, 21 male (42.5%) and 26 female interns (57.5%) participated. These communities were in three of the nine provinces, namely, Gauteng, Mpumalanga, and Limpopo. The characteristics of the organizations and interns in the sample are provided in Table 1.

**Table 1:** Characteristics of sample of organizations (NGOs) and sample (managers and trainees).

Characteristics of organisations as case studies									
Name	Location	Philosophy		Organizational structure and practice			Internal aspects^a^		External aspects^b^
Altus Sport	Urban	Youth empowerment, community development		Centralized program delivery, address community needs			Cascading leadership, develop youth leaders.		School-based, community engagement.
COTD	Urban and rural	Pathway development		Decentralized program delivery, psychosocial support			Serve most vulnerable—mentorship and resource provision		Center-based, support households in need
SCORE	Rural	Youth empowerment, sport participation		Decentralized program delivery, develop sport structures, and provide opportunities			Participation opportunities		School and community based
SOSA	Urban	Provide opportunities for special population		Decentralized program and standardized delivery			Provide opportunities for inclusion and address stigma		School and center-based with family engagement
Tidimalong	Urban	Provide social services and youth empowerment		Decentralized program delivery in addressing community needs			Support and care for most vulnerable		School and center-based delivery—networking
Characteristics of sample									
Managers	Position		Roles		Trainings		Program management		Delivery
	CEO (N)	Senior (N)	Strategic and leadership. Support and resource provision	Regular trainings – workshops and camps, on-site managerial support. Information resources and curricula/toolkits/manuals			Centralized quality control. Management support		Community- and individually centered models
	5	5							
Trainees	Age (N)	Gender (N)		Schooling (N)			Sport participation (N)		Currently unemployed (N)
	18–35 years	Male (N)	Female (N)	Matric	PS^c^—informal	PS—formal	Yes	No	All (47)
	All (47)	21	26	20	22	5	41	6	

NGO, nongovernmental organization; SOSA, Special Olympics South Africa; CEO, Chief Executive Officer; PS, post-school, COTD, Children of the Dawn.

^a^ Internal aspects—human resources (roles, training and teambuilding), outcomes, target of project, and expectations.

^b^ External aspects—stakeholder engagement, community engagement, and delivery.

^c^ Post-school—informal education include short courses or workshops not leading to a recognized qualification and formal education relates to recognized certificates, diplomas, and/or degrees.

### 2.4. Data analysis

Interviews and focus groups were transcribed verbatim following standardized coding procedures that entail line-by-line open coding, axial coding, and theme generation based on a grounded theory approach. Probing of issues during data collection contributed to in-context thematic development (by asking for clarifications and offering reflective summaries) to ensure semantic coherence (29).

Data collection and analysis were linked to the coding process that utilized constant comparison and reflection to ensure the semantic units identified are clustered for theme generation. The constant interfacing with data ensures the inductive development of meaningful constructs prior to integrate the data with deductive constructs (25).

## 3. Results

### 3.1. Case studies

In October 2021, a request for proposals was circulated to nonprofit making entities for the delivery of the Presidential Youth Employment Intervention (PYEF) as part of the Presidential Employment Stimulus (PES). The SSCNA was awarded a contract and started with the implementation by training five member organizations and representatives from SOSA. These sport-for-development organizations include Altus Sport, Children of the Dawn, SCORE, Tidimalong, and SOSA. SOSA made 4,000 placements for the implementation of their *So Fit* program at schools for Learners with Special Educational Needs (SLEN) by their athletes. In April 2022, the program commenced with training of interns and a rigorous MEL system to be implemented for the 6-month implementation period.

The National Youth Service program aims to address the “South Africa's chronic youth unemployment challenge” by requesting the placement of youth who would earn a minimum wage (stipend) while becoming more employable (30).

#### 3.1.1. Altus sport

The organization was founded in 1994 by two well-known athletes and qualified physical educators to provide meaningful education and training in sport and life skills. The organization played a leadership role during the 2010 FIFA World Cup in facilitating the Sport for Social Change Network and development or youth leadership in sport. By then, the focus changed from sport development to sport-for-development, although the organization continued to develop community sport structures in impoverished township areas by implementing a structured program delivered by youth leaders at schools and community centers (31). Altus Sport delivers an impactful gender-focused program, inclusive of entrepreneurial skills and building resilience at the individual and institutional levels (32). The interns were placed with the structured sport-for-development, reading, and community garden programs.

#### 3.1.2. Children of the Dawn (COTD)

Children of the Dawn is a nonprofit organization that was established in 2002 to focus on the support and strengthening of rural community initiatives by extending care and providing programs for orphans and vulnerable children in rural communities of South Africa (33). It received international recognition and attracted 23 funders by 2020 donating R8.81 million to offer programs and resources to eight rural communities situated in five of the nine provinces in South Africa (34). The organization follows a five-pronged approach through providing focused pathway programs to their beneficiaries.

The CARE program provides ongoing holistic support including nutritional and psychosocial support and access to safe spaces. The Children in Action Program provides stimulating, fun, and educational activities for children aged 5–13 years. My Future, My Responsibility exposes adolescents (14 years and older) to a variety of programs, while the Sport for Good program includes a variety of sport activities and camps. For beneficiaries who are at tertiary institutions, the organization offers Student Mentorship and Financial Assistance, creating opportunities for beneficiaries at the tertiary level to inspire and tutor younger beneficiaries.

#### 3.1.3. SCORE

SCORE was founded in 1991 when a team of American volunteers worked at schools in Khayelitsha (a township in Cape Town area) before handing the reigns to South Africans by 1993. International partners and global agencies supported, while SCORE attracted international volunteers to their sport-for-development initiatives in Southern Africa (then including Namibia, Zambia, and South Africa). In the early 2000s, the organization built and managed multiple multipurpose community sport facilities and provided sport, leadership, and life skill programs in 50 communities. Since 2015, they are an accredited service provider for delivering training and facilitation within the sector. The organization designs and manages the delivery of sport-for-development and educational programs, while delivering sport and life skill programs (including physical education classes) to schools and communities in response to local needs. They facilitate and support the creation of sustainable sport organizing structures in communities through a methodology that aims to deliver (i) participative assessment and planning; (ii) access, activity, and participation; (iii) skills and leadership training; (iv) organization building and development; and (v) developing and maintaining networks of support (35).

Another focus is to promote gender equity and transformation at the participation level in sport. In 2015, SCORE partnered again with the National Department of Sports and Recreation in delivering the School Sport Revival Program and supervised the training of 5,300 coaches in netball, volleyball, basketball, and tennis (Personal Interview with SCORE Manager, 35).

#### 3.1.4. Special Olympics South Africa (SOSA)

Since its establishment in 1968, Special Olympics as a global organization serves athletes with intellectual disabilities. In 1991, Special Olympics International founded and accredited Special Olympics South Africa. Since then, South Africa has competed regularly and featured about 57,000 athletes and unified partners and more than 3,000 coaches (personal interview with SOSA Manager). SOSA's mission is to “provide year-round sports training and athletic competition in a variety of Olympic–type sports for children and adults with an intellectual disability, giving them ongoing opportunities to develop physical fitness, demonstrate courage, experience joy and participate in the sharing of gifts, skills and friendship with their families, other Special Olympics athletes and the community.”

The organization prioritizes the development of athletes in 16 official sports and offer unified sports to break down the stereotypes of people with intellectual disabilities. Young athletes are offered an early childhood development program for children with and without intellectual disabilities between the ages of 2–7 years. Other development programs include Athlete Leadership, Healthy Athletes (HAs), Family Support Networks (FSNs), and Family Health Forums (FHFs) (36). Interns are placed at schools to facilitate the *So Fit* program and at entities for leadership development or centers for vocational training.

#### 3.1.5. Tidimalong

Tidimalong after Care Centre registered in April 2010 as a nonprofit organization with the Department of Social Development to bring about changes in the lives of destitute, orphans, vulnerable, and disadvantaged community members. Services offered include providing access to resources that will contribute to improved self-reliance, educational support and health-related education, physical activities (including sports), and stakeholder collaboration to access alternative care for orphan and vulnerable children (e.g., foster care) (personal interview with Tidimalong Manager).

Their services are linked to activities that aim to deliver positive health outcomes, positive academic and socioeconomic outcomes, access to public resources and services, increased resilience through counseling, and positive social outcomes through sport-for-development activities. The interns are placed at the head office for administrative work and at local community centers or educational institutions where services and programs are delivered to vulnerable populations in Winterveld communities situated in the Mabopane district.

### 3.2. Personal employment histories

A female intern from Tidimalong, who matriculated in 2012, underwent 1-year secretarial training (including computer skills) but was unsuccessful in obtaining wage employment. She explained

“Since I have been doing my schooling, I have never got a job for admin (sic) hence I am here at Tidimalong Primary School to do the admin and get the experience. This is my first job and my first contract. This will be my advantage as they want experience at least one to two years. I did not have that, and I saw this as an opportunity to gain experience” (focus group discussion).

The interns placed at SOSA are not only struggling with the stigma of intellectual disability but also did not obtain a qualification after completion of their schooling. The CEO of SOSA's quest is to create an ecosystem that would ensure that they would have a recognized body of evidence certified that would enhance their chances to enter the job market. Two- to three-year vocational training in carpentry, upholstery, welding, and sewing at the center visited in Thohoyandou provide some skills with which they may obtain local work or produce items of utility for their households.

Many interns studied but dropped out due to financial constraints, personal issues, found the coursework too difficult (e.g., physics, engineering), struggled with cultural adaptations when attending universities in cities, or obtained certificates or diplomas that did not make them competitive in a congested labor market. Overproduction of qualified candidates in humanities or in public administration, human resource management, general management, and marketing management provide limited opportunities for wage employment in an already saturated market. Many said that they need work-related experiences and “a strong CV.” Not being fluent in English is another stumbling block for jobseekers from rural areas.

After having given up on searching for employment in the formal sector, several opted for self-employment in the agricultural arena (running community gardens), education (starting a preschool or offering private homework lessons), and entertainment (e.g., singing, music, or drama) on demand.

Most consider themselves to be “lucky in obtaining several contracts” regardless of the short-term nature thereof. In some cases, it may be a contract lasting a few weeks or to a few months (“census” and “COVID-19 training” or “roadworks”) while it may last for several years (“handing out condoms in the community”). These contracts vary and candidates get “on the job training” within the sector—mostly being a government entity, business, or NGO. The following narratives attest to such vulnerable and the “underemployment” of interns.

 “I got my matric in 2013. I am a musician and I trained in classical (sic) like piano. I perform still even now. I work at an assistant teacher [as intern]. So, now only I do music and work at the Center. I get paid in the township and we do corporates [performances]” (male intern from Tidimalong, focus group discussion).

 I got my matric in 2016. The following year in 2017, I went to study business administration for three years and got a diploma in 2019. I specialized in supply chain management … then I did an in-service learning as part of the training and became self-employed in agriculture. The in-service time was one month only 20 hours … I want to be self-employed and do my own thing. I want to drive a truck and have it as a business” (female intern from Altus Sport, focus group discussion).

 “I left school on 2013 to 2015 I was Giyani Tech college for Generic Management. It is a certificate. I studied for three years and from 2018 to 2020, I was on contract with Rand Water for three years. I was doing maintenance of pipes. In 2021, I got a contract with department of Health doing malaria control. Yes, then this was for 12 months. Ja, then 2022 I got lucky, the Department of Greater Giyani under the Ward, I could do sports. I am working with the Councilor in sports … I am appointed there and here” (male intern from SCORE, focus group discussion).

From these accounts, it is apparent that local employment opportunities are limited, that not any qualification guarantees entry into the job market and that these youth count themselves “lucky” if they could obtain one or two multiyear contracts—“working in different jobs at the same time.” Further analysis shows a fragmented and disjunct vocational pathway with individuals accessing opportunities through personal connections (bonding and bridging social capital) and familiar opportunities in local settings (20).

### 3.3. Local embeddedness

All managers and interns believed that although it is possibly easier to find short-term employment in cities or bigger towns, many are driven home due to the cost of living. For young women, the family offers a supportive network when they become mothers, and in most households, the pooling of resources (including income and welfare funding) contributed to the survival of the collective (including direct and extended family). Sharing the cost of living enforced local positioning and considering entrepreneurial options within the local community.

Most are excluded in formal economic employment as schools may hire them on a 5-month contract as “teacher assistants,” “care workers” (Department of Health), or they may “work for a stipend” from an NGO when offering different services. None of the interns applied for low-paid (and stigmatized) jobs, such as domestic work (e.g., cleaning), farm labor, or working in mines. The cost of living and available networks of friends, family, and contacts with local schools (bridging social capital) enable access to employment opportunities (37).

### 3.4. A typology of work

A typology of work in terms of a positive trajectory unfolded. Most interns experienced “no work” (unemployed) at the low end (and most undesirable) of a continuum. Then, after some time of studying and dropping out or failing, not being able to find formal employment, they may enter volunteering, short contracts (if available), or starting up a venture for income generation—mostly as a family business or in collaboration with friends. Due to the recruitment requirements, none of the interns had formal employment experiences—inclusive of the graduates who were placed as interns with SOSA and Children of the Dawn.

The typology represents an overlay of existing work-related practices, ranging from being unemployed, having short-term contracts, access education, and training or transitioning into sustainable income generation in the formal or informal sector (e.g., entrepreneurship) (22).

### 3.5. Program benefits

All interns, except for Altus Sport and SOSA, referred to themselves as constituting the “organization on the ground.” This was particularly evident in rural communities where they have become “the face of the organization” and make community-level connections to deliver sport at local schools or community centers. The communities in Limpopo, where the SCORE program is offered, focus to establish sport clubs for soccer and netball. Altus Sport has a strong brand and high status at the schools where their trainees are placed. Regular engagements with schools where programs are offered provide youth leaders (inclusive of interns) with an overriding identity and brand. The interns of SOSA all work under direct supervision of local supervisors with SOSA overseeing the roll out of the So Fit program in a structured way. Personal benefits including personal development and “time management, discipline, and going the extra mile, acting as role models to children in the community” were cited by most interns. They must “log in and sync out” *via* their mobile phones that contributed to “acting like a professional.” For many, it was to form a “working person's identity” and to be able to earn an income that advanced their status as a coach, community worker, or service provider. This demonstrates the value of life skills and constructing new identities within a safe and supportive space where individuals bond and find recognition and acceptance (19). Especially SOSA interns were ecstatic when for the first time they “opened a bank account and got a bank card” and “showing their families and the community that they are worthy by finding paid work.”

All interns acknowledged the learning of technical (sport) skills and methodologies, content knowledge, as well as the “soft skills,” such as being able to communicate in English, speak in front of people, managing children, and experience an increase in self-knowledge and self-worth. For managers, it was mainly the “soft skills” that they value as contributing to increased employability because “earning a living through sport” is difficult, especially as sport is seen as rather trivial in impoverished households who were struggling to “put food on the table.”

### 3.6. Enablers and goal setting

For many, program implementation became a way to reflect on their employability status, the existing local job market, options for studies (including improving their matric marks to gain access to bursaries), and to consider future work-related options. Access to community networks, including schools and government departments, through delivering the program is seen as important for possible future contract work.

All interns working at a community facility or school value the bonding and support from their peers with whom they may “go into business” or who may support them when they are in need. In this way, being visible in the community may lead to possible employment opportunities at schools or from individuals whose children are attending the programs offered by them.

Access to physical resources (facilities and equipment) is more readily available at schools and the lack thereof necessitates some to buy “balls from our pockets” or “pay for transport for away-games.” There is a dire need for more and tangible benefits, knowledge, and methodologies that will enhance the employment status, such as getting a driver's license, computer courses, or administrative experience. Mentorship, guidance, and leadership from the parent organization is highly valued, particularly when successful professionals “give back,” as in the case of the Alumni program of Children of the Dawn.

## 4. Discussion

Although the interns were involved in the different organizations before their placement, the conversation of employability and employment directed their current engagement—giving them hope and purpose to gain experience, build a CV, develop responsibility, improve their communication skills, and have mock interviews (Children of the Dawn), and some use their income to improve their matric marks and save for education. Local organizations matched and placed interns to suit their skill set and interest. For instance, Tidimalong's youth who assisted with early childhood and social service delivery triggered individuals to further their studies in these fields. Vocational training for SOSA beneficiaries provides a direct avenue for income generation. It demonstrates a level of resilience and focus by engaging in forward-thinking of possible career pathways (13, 15).

Receiving a stipend alleviated the poverty at the individual and household level while countering the stigma of “uselessness and dependency” associated with unemployment. At all sites, interns communicated the importance of them contributing to their household's survival, facilitate siblings and their children, and invest in strengthening kinship ties for future support (38).

The rather innovative monitoring and evaluation system through digital methodology challenged the interns but contributed to the professional conduct relating to time management, ensuring implementation (service delivery) and stimulated creativity and problem-solving as essential soft skills to ensure optimal participation in sessions. In this way, teamwork and shared responsibilities foster peer-to-peer friendships and the anticipation that they may continue to work together in the future. Bonding social capital provided the building of trust and mutual caretaking (16, 8, 39).

The support from families and respect earned in the broader community contributed to individuals’ sense of self-worth and increased their visibility—with that, the potential for improved access to employment opportunities. In the absence of direct supervision, interns from SCORE in the remote villages of Limpopo focused on forming sport club structures and establish a sense of ownership that would bring recognition and opportunities that would reduce their own vulnerability status (9).

This opportunity of paid internship is for many others a chance to redirect their expectations, be realistic about opportunities within the local setting, yet find inspiration from other employed (and qualified) youth (alumni) who would reflect on their own pathways to employment. The potential to find bursaries, engage in social enterprises, finding opportunities for “job-shadowing” and gain valuable lessons of pitfalls and strategies for becoming employable and finding employment, were not readily available.

The managers as a cohort had to adapt existing programs to address youth unemployability but lack the capacity and resources to ensure that most will transition into employment. Although the interns bonded as a group and generate bridging social capital at the organizational level, limited linking social capital exist to ensure vertical linkages with other stakeholders to absorb the youth in the existing job market.

Being engaged in a sport-for-development program provided youth with a high level of soft skill acquisition transferable to a working environment. The challenge remained to ensure an appropriate skill set to find employment in sport or other sectors.

Youth work within the sport-for-development sector may enhance youth employment when key mechanisms are in place. The latter refers to the development of soft skills, integration into an ecosystem of related work within and across sport and other sectors, as well as pathway assistance. A robust MEL system, consultation, and dissemination of findings to guide strategic planning inform a transformative agenda for all stakeholders. Contextual realities require nuanced and diverse strategies in addressing youth unemployment with the recognition that one size does not fit all.

## Data Availability

The datasets presented in this article are not readily available because it is qualitative data and I would not have “raw data” used by others as the research participants did not give permission and informed consent was signed to use the narratives only by me and for publication purposes (and anonymous) only. The names of the organizations and their profiles have been approved by the Presidents/CEOs. Requests to access the datasets should be directed to the corresponding author.
